# Effect of Chronic Pediatric Heart Conditions on Family Dynamics in Saudi Arabia: A Cross-Sectional Study

**DOI:** 10.7759/cureus.98279

**Published:** 2025-12-01

**Authors:** Aljuhara A Alsuayyid, Abdullah S Alosayl, Zahra H Al Dawood, Eman A Alzayer, Saleh S Alenazi, Yahya Almashham

**Affiliations:** 1 Pediatric Cardiology, King Fahad Medical City, Riyadh, SAU

**Keywords:** chronic heart disease (chd), coping mechanisms, emotional stress, family centered care, family dynamics, kingdom of saudi arabia (ksa), . pediatric cardiology, pediatric congenital heart disease

## Abstract

Introduction: Chronic pediatric heart diseases significantly affect family dynamics, therefore affecting caregivers' emotional, financial, and social spheres of life. Understanding how healthcare systems and family structures differ in Saudi Arabia helps us improve support systems since they directly affect families. This study investigated how chronic pediatric cardiac diseases affected family dynamics and what elements supported caregivers' stress and coping strategies.

Methods: Between February and March 2025, a cross-sectional survey was conducted at King Fahad Medical City (KFMC), Riyadh, Saudi Arabia. Through a self-administered survey, data were gathered from parents or main caregivers of children with chronic cardiac diseases, including congenital and acquired heart conditions. The aspects of family life evaluated in the survey were emotional effects, financial difficulty, social dynamics, and coping mechanisms. Data were analyzed utilizing thematic analysis and descriptive statistics.

Results: One hundred families were included in the study; most of their children, 59 (59%), were diagnosed with septal defects. Many of the households had not undergone any significant treatment, 59 (59%). With 56 (56%) caregivers reporting stress caused by the child's condition and 22 (22%) suffering financial hardship, results showed major emotional and economic difficulties. Moreover, 63 (63%) families reported receiving emotional support from medical professionals. Still, 63 (63%) parents looked for more specialized mental health assistance. Regarding the effects of the child's condition and variables like gender, nationality, or main diagnosis, no major demographic differences were discovered.

Conclusion: In Saudi Arabia, chronic childhood cardiac problems greatly affect family dynamics and provide major emotional, financial, and social difficulties. Families stated that they got some healthcare assistance; therefore, more psychological resources and all-encompassing support networks are required to solve the emotional and economic burden on caregivers. The results emphasize the importance of including family-centered care in pediatric cardiology clinics and encouraging more developed support networks for families coping with chronic diseases.

## Introduction

Chronic pediatric heart conditions, such as congenital heart defects, affect a significant number of children; these conditions can vary in severity and may require different treatments for each child [1,2]. While medical advances have improved survival rates, 95% of these children reach adulthood due to the success of modern treatment options [3]. Chronic pediatric heart conditions pose significant challenges for the child and their families. Parents of children with congenital heart disease (CHD) are more susceptible to psychological and social distress [4], high levels of parenting stress, poor sleep, and elevated psychological maladjustment [5]. The challenges the condition presents can affect the family at various stages, from diagnosis through childhood. The diagnosis of CHD, paired with subsequent surgical and interventional treatments and prolonged hospital stays, causes acute psychological distress and can lead to post-traumatic stress disorder (PTSD) [6]. The ongoing care and uncertainty often create emotional, financial, and social strains on family members. These strains can significantly impact their roles, relationships, and coping mechanisms, highlighting the profound effect of these conditions on family dynamics. Family socioeconomic status is an essential factor associated with health-related quality of life (HRQOL) in patients with critical congenital heart disease [7]. In addition to higher levels of anxiety and depression in CHD parents, they reported lower confidence in their abilities to parent. Clinicians must identify at-risk parents early to provide them with systematic support [6]. The National Institute for Health and Care Excellence (NICE) recommends that children with CHD and their families may need support and psychological help to cope with the emotional challenges that come with managing this condition [3]. Despite a wealth of research on medical outcomes, there is limited exploration of how these conditions affect family dynamics in Saudi Arabia. This study aims to fill this gap by examining how chronic pediatric heart conditions influence family interactions and overall functioning. The findings will enhance our understanding of these conditions and provide valuable insights to help healthcare providers, social workers, and policymakers create better support systems for affected families.

## Materials and methods

This study employed a cross-sectional survey design, using a questionnaire to gather data from families of children with chronic heart conditions receiving treatment at King Fahad Medical City (KFMC). Data collection took place between February 2025 and March 2025. Families attending the pediatric cardiology department as inpatients and outpatients were approached to participate.

The target population for the study included parents or primary caregivers of children diagnosed with chronic heart conditions at KFMC. The inclusion criteria specified that families of children under 14 years who had a diagnosed chronic heart condition (e.g., congenital heart defects or acquired heart diseases) were eligible for participation. Both parents or caregivers were invited to participate, with the option of one or both completing the questionnaire.

The exclusion criteria included families of children not receiving care for heart conditions during the study period, syndromic patients, and children with non-cardiac diseases.

Data were collected through a self-administered questionnaire designed to assess various aspects of family dynamics, including the emotional, financial, and social impacts of caring for a child with a chronic heart condition. The questionnaire included both close-ended and open-ended questions to provide a comprehensive understanding of the family experience. Key areas of inquiry included emotional impact, social and relationship dynamics, financial burden, coping strategies, and the availability and effectiveness of support systems.

Close-ended questions provided quantitative data on stress levels, relationship changes, and financial strain, while open-ended questions allowed personal narratives and insights into family adjustment and coping mechanisms.

Following ethical approval by KFMC, families were approached during routine visits to the pediatric cardiology department. Parents and caregivers were fully informed about the purpose of the study, the voluntary nature of participation, and the confidentiality of the data. Those who agreed to participate were provided with the questionnaire, which they could complete at their own pace, either in the hospital or at home. The completed questionnaires were expected to be submitted by the end of the study period in March 2025.

The collected data were analyzed using descriptive statistics to summarize demographic information and assess the impact of chronic pediatric heart conditions on family dynamics. Frequencies, percentages, and mean scores were calculated for close-ended questions. Open-ended responses were subjected to thematic analysis, which involved identifying recurring themes and patterns related to family experiences and coping strategies. This analysis aimed to provide a deeper understanding of the emotional, social, and financial challenges faced by families.

The study adhered to ethical guidelines, ensuring all participants provided informed consent. Participants were made aware of the study's purpose, the voluntary nature of their involvement, and their right to confidentiality. All personal identifying information was kept confidential and securely stored in compliance with ethical and social research standards.

## Results

As shown in Table 1, the study included 100 children with chronic heart conditions, with a mean age of 49.15 months (SD = 52.7). Males represented 58% (58) of the sample, while females accounted for 42% (42). Most of the children were Saudi nationals (92%), and the most common primary diagnosis was septal defects (59%), followed by right heart lesions (10%) and single ventricle conditions (10%). More than half of the children (59%) had not undergone any interventions, whereas 27 (27%) and 10 (10%) underwent catheterization. The average number of siblings was 3.02 (SD = 2.83), indicating that most children came from moderately sized families.

**Table 1:** Demographic characteristics of children with chronic heart conditions

		Count	Column N %
Age	Mean (SD)	49.15 (52.7)	
Gender of child	Male	58	58.0%
	Female	42	42.0%
Nationality	Saudi	92	92.0%
	Non-Saudi	8	8.0%
Primary diagnosis	Septal defects	59	59.0%
	Right heart lesions	10	10.0%
	Left heart lesions	4	4.0%
	Coronary artery anomalies	2	2.0%
	Electrophysiologic conditions	6	6.0%
	Single ventricle	10	10.0%
	CoA	3	3.0%
	PDA	6	6.0%
Intervention	Not done	59	59.0%
	Surgery	27	27.0%
	Catheterization	10	10.0%
	Both	4	4.0%
Number of surgeries	Mean (SD)	0.31 (0.48)	
Number of siblings	Mean (SD)	3.02 (2.83)	

Table 2 summarizes the demographic characteristics of parents and caregivers. Most respondents were mothers (62%), followed by fathers (36%), with an average maternal age of 35.6 years (SD = 8.2) and paternal age of 40.3 years (SD = 9.8). Most fathers (41%) and mothers (55%) hold a bachelor's degree. Employment status varied, with 33% of fathers working in government jobs and 28% in the private sector, while most mothers (76%) were housewives. Almost all children (93%) live with both parents, and most parents were married (93%). Most children were diagnosed during the neonatal period (64%), reflecting early detection and management of cardiac conditions.

**Table 2:** Demographic characteristics of parents and caregivers

		Count	Column, N%
Relation to child	Mother	62	62.0%
	Father	36	36.0%
	Other (Aunt, Brother)	2	2.0%
Father's age	Mean (SD)	40.3 (9.8)	
Father's education	NA	3	3.0%
	Illiterate	2	2.0%
	Less than high school	11	11.0%
	High school/Diploma	37	37.0%
	Bachelor	41	41.0%
	Master/PhD	6	6.0%
Father's job	NA	3	3.0%
	None	2	2.0%
	Private sector	28	28.0%
	Government	33	33.0%
	Freelancer	7	7.0%
	Military	23	23.0%
	Retired	4	4.0%
Father alive	No	2	2.0%
	Yes	98	98.0%
Mother's age	Mean (SD)	35.6 (8.2)	
Mother's education	NA	2	2.0%
	Illiterate	3	3.0%
	Less than high school	8	8.0%
	High school/Diploma	32	32.0%
	Bachelor	55	55.0%
	Master/PhD	0	0.0%
Mother's job	NA	2	2.0%
	None/Housewife	76	76.0%
	Private sector	6	6.0%
	Government	5	5.0%
	Teacher	8	8.0%
	Nurse	3	3.0%
Mother alive	No	1	1.0%
	Yes	99	99.0%
The child lives with	Both parents	93	93.0%
	Mothers	6	6.0%
	Other (Aunt)	1	1.0%
Parents status	Married	93	93.0%
	Divorced	5	5.0%
	Widow	1	1.0%
	Both dead	1	1.0%
Time of diagnosis	Antenatal	5	5.0%
	Neonatal period	64	64.0%
	Infancy	18	18.0%
	After 1st year	13	13.0%

Table 3 illustrates the impact of chronic pediatric heart conditions on family dynamics. A significant proportion of parents reported experiencing stress due to the child’s condition, with 56% experiencing stress sometimes and 31% always. Mood changes since the diagnosis were also common, with 60% of respondents experiencing mood changes sometimes. The relationship with the partner was most often reported as unaffected (76%), although 20% of parents reported occasional negative effects. Financial strain due to treatment costs was a notable issue, with 50% of families reporting financial strain sometimes and 22% always. Regarding self-care, 54% of families sometimes struggled to find time for themselves. A significant number of parents (59%) experienced guilt as caregivers, with 37% reporting it sometimes. Disrupted sleep due to the child's condition was a concern for 44% of families. Support from healthcare professionals was highly valued, with 63% reporting always receiving emotional support. Despite this, a significant number of parents (63%) sought professional support for mental health. Managing the child’s needs with confidence was reported by 79% of respondents, reflecting high levels of coping confidence. Additionally, 64% of families reported that the child’s condition affected future family planning.

**Table 3:** Impact of chronic pediatric heart conditions on family dynamics

	Never		Sometimes		Always	
	Count	Row (N%)	Count	Row (N%)	Count	Row (N%)
Stress due to the child's condition	13	13.0%	56	56.0%	31	31.0%
Mood changes since diagnosis	27	27.0%	60	60.0%	13	13.0%
Negative effect on the relationship with the partner	76	76.0%	20	20.0%	4	4.0%
Strain due to the financial cost of treatment	50	50.0%	28	28.0%	22	22.0%
Time for self-care activities	28	28.0%	54	54.0%	18	18.0%
Experiencing guilt as a caregiver	59	59.0%	37	37.0%	4	4.0%
Disrupted sleep due to the child's condition	35	35.0%	44	44.0%	21	21.0%
Emotional support by healthcare professionals	10	10.0%	27	27.0%	63	63.0%
Seeking professional support	63	63.0%	29	29.0%	8	8.0%
Managing a child's needs confidently	0	0.0%	21	21.0%	79	79.0%
Impact on family/future planning	64	64.0%	23	23.0%	13	13.0%
Support from extended family	12	12.0%	26	26.0%	62	62.0%
Frequent hospital admissions	55	55.0%	36	36.0%	9	9.0%
Developmental impact	53	53.0%	30	30.0%	17	17.0%

**Figure 1:**
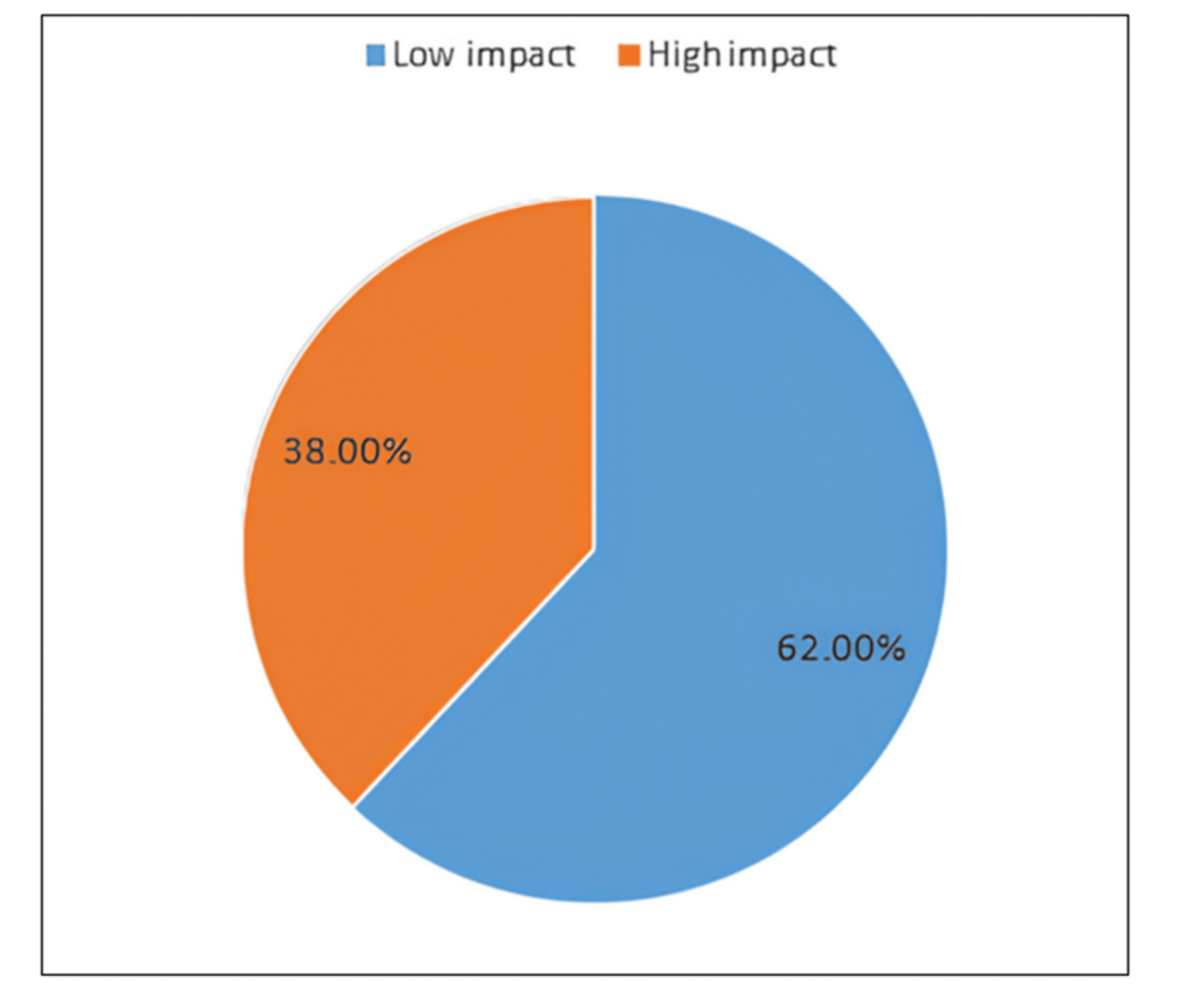
Impact of chronic pediatric heart conditions on family dynamics in Saudi Arabia

Figure 1 visually represents the overall impact of chronic pediatric heart conditions on family dynamics in Saudi Arabia. It shows that 62% of families reported a low impact, while 38% reported a high impact, indicating a substantial portion of families affected by the condition.

Table 4 explores the relationship between the impact of the child's condition and various demographic factors. The analysis revealed no significant difference in the effects based on the child’s gender (p = 0.987) or nationality (p = 0.137). Regarding the primary diagnosis, children with right heart lesions and coronary artery anomalies showed a higher proportion of high-impact responses (80% and 100%, respectively); however, these differences were not statistically significant (p = 0.111). The type of intervention also showed no significant association with the reported level of impact (p = 0.548). Similarly, the father’s education level and occupation, as well as the mother's education level, did not show significant differences in reported impact, with p-values ranging from 0.083 to 0.514. Family status was also not significantly associated with the impact level. This was reflected in the responses of children living with both parents (p = 0.245), divorced parents (p = 0.399), and children of widowed parents (p = 0.199).

**Table 4:** Association between the impact of the child's conditions and demographic factors

		Impact				
		Low impact		High impact		P-value
		Count	Row (N%)	Count	Row (N%)	
Gender of child	Male	36	62.1%	22	37.9%	0.987
	Female	26	61.9%	16	38.1%	
Nationality	Saudi	59	64.1%	33	35.9%	0.137
	Non-Saudi	3	37.5%	5	62.5%	
Primary diagnosis	Septal defects	39	66.1%	20	33.9%	0.111
	Right heart lesions	2	20.0%	8	80.0%	
	Left heart lesions	3	75.0%	1	25.0%	
	Coronary artery anomalies	2	100.0%	0	0.0%	
	Electrophysiologic conditions	3	50.0%	3	50.0%	
	Single ventricle	6	60.0%	4	40.0%	
	CoA	3	100.0%	0	0.0%	
	PDA	4	66.7%	2	33.3%	
Intervention	Not done	39	66.1%	20	33.9%	0.548
	Surgery	14	51.9%	13	48.1%	
	Catheterization	7	70.0%	3	30.0%	
	Both	2	50.0%	2	50.0%	
Father's education	NA	2	66.7%	1	33.3%	0.473
	Illiterate	0	0.0%	2	100.0%	
	Less than high school	8	72.7%	3	27.3%	
	High school/Diploma	22	59.5%	15	40.5%	
	Bachelor	27	65.9%	14	34.1%	
	Master/PhD	3	50.0%	3	50.0%	
Father's job	NA	2	66.7%	1	33.3%	0.514
	None	1	50.0%	1	50.0%	
	Private sector	16	57.1%	12	42.9%	
	Government	23	69.7%	10	30.3%	
	Freelancer	3	42.9%	4	57.1%	
	Military	16	69.6%	7	30.4%	
	Retired	1	25.0%	3	75.0%	
Father alive	No	1	50.0%	1	50.0%	0.724
	Yes	61	62.2%	37	37.8%	
Mother's education	NA	1	50.0%	1	50.0%	0.083
	Illiterate	0	0.0%	3	100.0%	
	Less than high school	3	37.5%	5	62.5%	
	High school/Diploma	20	62.5%	12	37.5%	
	Bachelor	38	69.1%	17	30.9%	
	Master/PhD	0	0.0%	0	0.0%	
Mother's job	NA	1	50.0%	1	50.0%	0.371
	None/Housewife	46	60.5%	30	39.5%	
	Private sector	2	33.3%	4	66.7%	
	Government	4	80.0%	1	20.0%	
	Teacher	6	75.0%	2	25.0%	
	Nurse	3	100.0%	0	0.0%	
Mother alive	No	0	0.0%	1	100.0%	0.199
	Yes	62	62.6%	37	37.4%	
The child lives with	Both parents	57	61.3%	36	38.7%	0.245
	Mothers	5	83.3%	1	16.7%	
	Other (Aunt)	0	0.0%	1	100.0%	
Parents' status	Married	57	61.3%	36	38.7%	0.399
	Divorced	4	80.0%	1	20.0%	
	Widow	1	100.0%	0	0.0%	
	Both dead	0	0.0%	1	100.0%	

## Discussion

The findings of this research offer valuable insights into the demographic profile and the impact of chronic pediatric cardiac conditions on family dynamics in Saudi Arabia. The predominance of male patients (58; 58%) aligns with earlier research reporting a higher incidence of congenital heart abnormalities among males [8,9]. A large proportion of children were Saudi nationals, reflecting the demographic composition of the patient population served by KFMC. Consistent with international literature showing that septal defects are among the most prevalent congenital cardiac anomalies, septal defects were the most frequent primary diagnosis in this cohort (59; 59%), highlighting their common occurrence and the continuing need for targeted clinical and supportive intervention [10,11].

With regard to treatment patterns, the fact that a large proportion of children (59; 59%) had not undergone any intervention may reflect the variability in the severity of heart conditions and the availability of alternative management options, such as medication or regular monitoring [12]. Meanwhile, the number of children requiring invasive procedures is reflected in the relatively high proportion who underwent surgery (27; 27%) or catheterization (10; 10%) [13]. Medical procedures used to treat difficult pediatric heart diseases are typically performed in specialized hospitals. The mean number of surgeries per child (0.3) and the average number of siblings (3.02) are consistent with earlier research, reflecting moderate family sizes in the region and variability in surgical needs depending on each child’s condition and response to treatment [14].

A significant concern identified in this study was the financial burden, with 22 (22%) families reporting a high financial strain. This is consistent with global literature showing that families managing chronic pediatric illnesses often face substantial economic challenges, particularly in healthcare systems with mixed public-private structures [15].

Additionally, 54 (54%) families occasionally struggled to find time for self-care, and 44 (44%) reported disrupted sleep. These findings underscore the personal toll caregiving takes on parents, a topic widely documented in the caregiving literature [16-18].

Emotional support from medical professionals was reported as consistently helpful, with 63 (63%) respondents saying they always received emotional support. This underscores the importance of comprehensive care that addresses both the medical and emotional needs of the child and family [19]. However, despite receiving emotional support, 63 (63%) parents also sought professional mental health services, indicating that the existing support system may fall short of fully meeting the psychological needs of caregivers [20].

Despite these challenges, a significant number of parents reported high confidence in managing their child's condition, 79 (79%). This reflects the resilience and adaptive coping strategies frequently observed among parents of children with chronic illnesses [21].

Statistical analysis revealed no significant differences in the reported impact based on the child’s gender or nationality, suggesting that the burden of chronic pediatric heart conditions is broadly similar across these variables. However, families of children diagnosed with coronary artery anomalies or right heart lesions reported a higher proportion of severe impact, consistent with the more complex nature of these conditions and the need for intensive treatment. These cases may place increased emotional, financial, and logistical strain on families [22,23]. Interestingly, the type of intervention did not significantly influence the perceived impact, suggesting that the other factors, such as the child’s overall health status and the family’s coping mechanisms, play a more critical role in shaping family dynamics.

This study has several limitations. First, the relatively small sample size of 100 families makes it difficult to apply the findings to all families of children with chronic heart conditions in Saudi Arabia. Second, the cross-sectional design provides information from one point in time and cannot show how families' experiences or stress levels may change over time. Third, the study was conducted at a single tertiary care center, so the results may not reflect experiences in other regions or health care settings with different socioeconomic or cultural backgrounds. Finally, the reliance on self-reported data introduces the possibility of response bias, as participants may underreport or overreport emotional, financial, or social challenges. Despite these limitations, this study provides valuable preliminary insights into the emotional, social, and financial challenges faced by families of children with chronic heart conditions in Saudi Arabia. These findings highlight areas of further investigation and may serve as a foundation for future research using larger, multicenter, and longitudinal designs.

## Conclusions

In essence, the findings of this research highlight that chronic pediatric heart conditions have significant emotional, social, and financial impacts on families in Saudi Arabia. These results underscore the need for more holistic healthcare approaches that encompass both medical treatment and comprehensive family support services. Such services should include caregiver education, stress management strategies, and a financial assistance program to help families navigate the challenges of caregiving.

Further research is needed to explore the long-term effects of these conditions on family relationships and to evaluate the effectiveness of support programs designed to assist families throughout the treatment journey.

## Appendices

Effect of chronic pediatric heart conditions on family dynamics in Saudi Arabia 

Data collection sheet

1. Age:

2. Gender: Male          Female

3. Nationality: Saudi       Non-Saudi

4. Primary diagnosis:

Septal defect: ASD, VSD, AVSD, APW, PAPVDs

Right heart lesions: TOF + PA, TOF absent PV, PA + IVS, Ebstein anomalies, TS, PS, PAs stenosis, DCRV.

Left heart lesions: AS, AI, MS, MVP, BAV, other outflow tract abnormalities in the LV.

Single ventricle.

Conotruncal anomalies: TGA, DORV, Truncus arteriosus.

Coronary artery anomaly.

CoA.

IAA.

PDA.

Vascular ring and sling.

Aortic root/Ascending aorta dilatation.

Electrophysiologic conditions.

Others: KD, pericarditis, endocarditis, aneurysm, cardiac tumor, PHT, myocardial infarction.

Post-surgery    ○      Intervention    ○         None   ○        Number, if any:

5. Relation to the child: Mother ○        Father   ○        Others (please specify):

Father: Age:         Level of education:         Job:        Alive (Yes/No)

Mother: Age:         Level of education:         Job:        Alive (Yes/No)

6. The child lives with: Father ○        Mother ○        Both   ○        Others (please specify):

7. Parents' status: Married  ○        Separated ○

8. Time of diagnosis: Antenatal ○        Neonatal period (in the first month) ○        Infancy (first year of life) ○      After 1st year ○

9. Number of siblings:

10. I experience stress related to my child’s heart condition.

Always ○        Sometimes ○        Never ○

11. I have noticed mood changes or emotional distress since my child’s diagnosis.

Always ○        Sometimes ○        Never ○

12. My relationship with my partner has been negatively affected by the caregiving responsibilities.

Always ○        Sometimes ○        Never ○

13. The financial cost of treatment has created strain on our family.

Always ○        Sometimes ○        Never ○

14. I find it difficult to make time for self-care activities (e.g., rest, relaxation, and hobbies).

Always ○        Sometimes ○        Never ○

15. I experience guilt in my role as a caregiver.

Always ○        Sometimes ○        Never ○

16. My sleep is often disrupted due to my child’s condition.

Always ○        Sometimes ○        Never ○

17. I feel emotionally supported by healthcare professionals involved in my child’s care.

Always ○        Sometimes ○        Never ○

18. I actively seek professional support (e.g., therapy or counseling) to cope with stress.

Always ○        Sometimes ○        Never ○

19. I feel confident in managing my child’s medical and emotional needs.

Always ○        Sometimes ○        Never ○

20. My child’s condition has impacted our future family plans or relationships with other children.

Always ○        Sometimes ○        Never ○

21. I feel supported by extended family members in managing caregiving responsibilities.

Always ○        Sometimes ○        Never ○

22. My child requires frequent hospital admissions due to their heart condition.

Always ○        Sometimes ○        Never ○

23. I believe my child’s physical or developmental progress has been affected by their medical condition.

Always ○        Sometimes ○        Never ○
